# 1,2-Dimeth­oxy-4-methyl-3-[(*S*)-*p*-tolyl­sulfin­yl]benzene

**DOI:** 10.1107/S1600536811041420

**Published:** 2011-10-12

**Authors:** Virginia M. Mastranzo, José Luis Olivares, Rubén Sánchez-Obregón, Francisco Yuste, Rubén A. Toscano

**Affiliations:** a Instituto de Química, UNAM, Circuito Exterior, Ciudad Universitaria, Delegación Coyoacán, CP 04510, México, DF, Mexico

## Abstract

In the title compound, C_16_H_18_O_3_S, the dihedral angle between the benzene rings is 75.48 (8)°. The absolute configuration at the stereogenic S-atom center was determined as *S*. The crystal structure is stabilized by inter­molecular C—H⋯O contacts.

## Related literature

For related sulfoxides, see: Brondel *et al.* (2010 ▶); Fuller *et al.* (2009 ▶). The title compound was prepared as a starting material for the synthesis of the tetra­hydro­protoberberine alkaloids (*S*)-(−)-tetra­hydro­palmatine and (S)-(−)-canadine following a synthetic strategy similar to that used for the synthesis of (*S*)-(−)-xylopinine (Mastranzo *et al.*, 2011 ▶). 
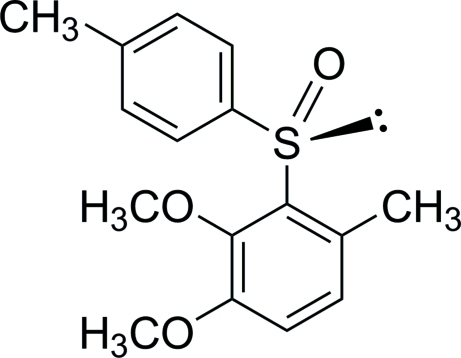


## Experimental

### 

#### Crystal data

C_16_H_18_O_3_S
*M* *_r_* = 290.36
Orthorhombic, 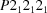

*a* = 7.4170 (6) Å
*b* = 8.5406 (7) Å
*c* = 24.0980 (19) Å
*V* = 1526.5 (2) Å^3^
*Z* = 4
Mo *K*α radiation
μ = 0.22 mm^−1^
*T* = 298 K
0.38 × 0.37 × 0.31 mm

#### Data collection

Bruker SMART APEX CCD diffractometer
Absorption correction: multi-scan (*SADABS*; Sheldrick, 2000 ▶) *T*_min_ = 0.922, *T*_max_ = 0.942
20418 measured reflections
3645 independent reflections
3380 reflections with *I* > 2σ(*I*)
*R*_int_ = 0.028

#### Refinement

*R*[*F* ^2^ > 2σ(*F* ^2^)] = 0.039
*wR*(*F* ^2^) = 0.099
*S* = 1.06
3645 reflections
185 parameters
H-atom parameters not refined
Δρ_max_ = 0.25 e Å^−3^
Δρ_min_ = −0.13 e Å^−3^
Absolute structure: Flack (1983 ▶), 1534 Friedel pairs
Flack parameter: 0.01 (6)

### 

Data collection: *SMART* (Bruker, 1999 ▶); cell refinement: *SAINT* (Bruker, 2006 ▶); data reduction: *SAINT*; program(s) used to solve structure: *SHELXS97* (Sheldrick, 2008 ▶); program(s) used to refine structure: *SHELXL97* (Sheldrick, 2008 ▶); molecular graphics: *SHELXTL* (Sheldrick, 2008 ▶); software used to prepare material for publication: *SHELXL97*.

## Supplementary Material

Crystal structure: contains datablock(s) I, global. DOI: 10.1107/S1600536811041420/bt5660sup1.cif

Structure factors: contains datablock(s) I. DOI: 10.1107/S1600536811041420/bt5660Isup2.hkl

Supplementary material file. DOI: 10.1107/S1600536811041420/bt5660Isup3.cdx

Supplementary material file. DOI: 10.1107/S1600536811041420/bt5660Isup4.cml

Additional supplementary materials:  crystallographic information; 3D view; checkCIF report

## Figures and Tables

**Table 1:** Hydrogen-bond geometry (Å, °)

*D*—H⋯*A*	*D*—H	H⋯*A*	*D*⋯*A*	*D*—H⋯*A*
C8—H8*B*⋯O3^i^	0.96	2.58	3.366 (3)	139
C14—H14⋯O1^ii^	0.93	2.59	3.457 (2)	155
C15—H15⋯O2^ii^	0.93	2.55	3.3886 (18)	150
C12—H12⋯O3^iii^	0.93	2.56	3.406 (2)	152

Symmetry codes: (i) 
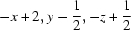
; (ii) 
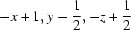
; (iii) 
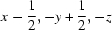
.

**Fig. 1.**
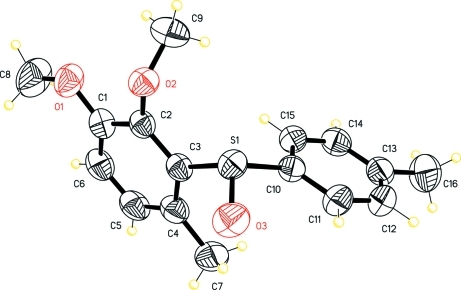
The molecular structure of (I), with atom labels and 50% probability displacement ellipsoids for non-H atoms.

**Fig. 2.**
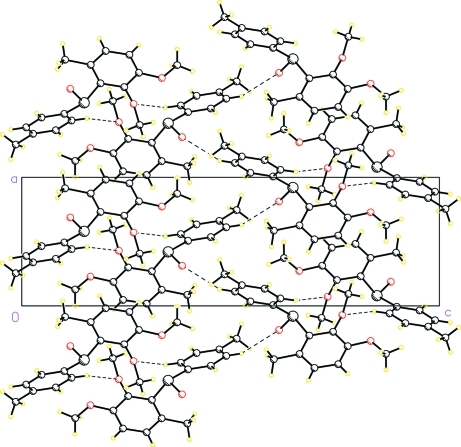
The packing of the title compound, viewed down the b axis, showing one layer of molecules connected by C—H···O—C and C—H···O—-S hydrogen bonds (dashed lines).

## supplementary crystallographic information

### Comment

The compound (*S*)-1,2-dimethoxy-4-methyl-3-(*p*-tolylsulfinyl)benzene was prepared as starting material for the synthesis of the tetrahydroprotoberberine alkaloids (*S*)-(-)-tetrahydropalmatine and (S)-(-)-canadine following a synthetic strategy similar to that used for the synthesis of (S)-(-)-xylopinine (Mastranzo *et al.*, 2011). The absolute configuration of the levorotatory 1,2-dimethoxy-4-methyl-3- (*p*-tolylsulfinyl)benzene (I) was determined by X-ray structure analysis. Figure 1 clearly shows that has the (S) configuration at the stereogenic S1 center. The geometry at S1 conforms to that found for related *p*-tolyl sulfinyl derivatives, it adopts an asymmetric propeller-like conformation having the corresponding C*_ortho_*—C*_ipso_*—S—O dihedral angles equal to -53.11 (15) and -28.07 (16)°. The dihedral angle defined by the least-squares planes of the aromatic rings is 75.48 (8)°. In the structure there are some intermolecular C—H···O contacts.

### Experimental

1-(3,4-Dimethoxyphenyl)-*N*,*N*-dimethylmethanamine

To a stirred solution of dimethylamine hydrochloride (6.4 g, 78.48 mmol) in MeOH (100 ml) KOH (1.2 g, 21.38 mmol) was added. When the pellets were completely dissolved, 3,4-dimethoxybenzaldehyde (10.0 g, 60.17 mmol) was added in one portion. The resulting suspension was stirred at room temperature for 15 min. Then, a solution of NaBH_3_CN (1.5 g, 23.87 mmol) in MeOH (15 ml) was added dropwise over 30 min. After the addition was complete, the suspension was stirred for 5 h. Potassium hydroxide (15 g) was then added, and stirring was continued until the pellets were completely dissolved. The reaction mixture was filtered with suction, and the filtrate was reduced to approximately 50 ml with a rotary evaporator while the bath temperature was kept below 40°C. To this concentrate EtOAc (100 mL) and brine (25 ml) were added and the layers were separated. The aqueous layer was extracted with EtOAc (2x50 ml). The combined organic layers were washed with brine (10 ml), dried over anhydrous potassium carbonate and concentrated. The residue was purified by flash chromatography (98:2 EtOAc/Et_3_N) to obtain a pale yellow liquid (8.21 g, 70% yield).

(*S*)-1-[3,4-Dimethoxy-2-(*p*-tolylsulfinyl)phenyl]-*N*,*N*-dimethylmethanamine

To a stirred solution of 1-(3,4-dimethoxyphenyl)-*N*,*N*-dimethylmethanamine (1.6 g, 8.20 mmol) in THF (30 ml) cooled at 0–5°C a 2.3 *M* solution in hexanes of n-BuLi (4.1 ml, 9.02 mmol) was added dropwise. After 2 h, a solution of (*S*)-menthyl *p*-toluenesulfinate (2.9 g, 9.84 mmol) in THF (10 ml) was added *via* cannula. The resulting mixture was stirred at room temperature for 4 h. Then, the reaction mixture was quenched with of saturated NH_4_Cl (10 ml) and extracted with EtOAc (3 *x* 20 ml). The combined organic layers were dried over Na_2_SO_4_ and evaporated. The residue was purified by flash chromatography (99:1 EtOAc/Et_3_N) to give a pale yellow oil (2.4 g, 88% yield), [α]_D_ -16.5 (c 1, CHCl_3_).

(*S*)-1-(Chloromethyl)-3,4-dimethoxy-2-(*p*-tolylsulfinyl)benzene

To a slurry of (*S*)-1-[3,4-dimethoxy-2-(*p*-tolylsulfinyl)phenyl]-*N*,*N*- dimethyl methanamine (4.02 g, 11.98 mmol, 1 equiv) and K_2_CO_3_ (2.64 g, 19.17 mmol, 1.6 equiv) in THF (10 ml) cooled at -78°C ethyl chloroformate (2.08 g, 19.17 mmol, 1.6 equiv) was added. The reaction was stirred at room temperature for 12 h. The resulting mixture was quenched with water (10 ml) and extracted with EtOAc (3x15 ml). The combined organic layers were dried over Na_2_SO_4_ and evaporated. The residue was purified by flash chromatography (70:30 hexane/EtOAc) to give a pale yellow solid (2.66 g, 68% yield), mp 77–78°C, [α]_D_ -132.4 (c 1, CHCl_3_).

(*S*)-1,2-Dimethoxy-4-methyl-3-(*p*-tolylsulfinyl)benzene (I)

A mixture of (*S*)-1-(chloromethyl)-3,4-dimethoxy-2-(*p*-tolylsulfinyl) benzene (1.72 g, 5.29 mmol) and NaBH_4_ (1.2 g, 31.77 mmol) in THF (30 ml) was heated at reflux for 18 h. The reaction mixture was quenched with Na_2_SO_4_.10H_2_O and filtered through Celite. The filtrate was evaporated under vacuum and the residue purified by flash chromatography (80:20 hexane/EtOAc) to obtain a white solid (1.03 g, 67%), mp 127–128°C, [α]_D_ -198.2 (c 1, CHCl_3_).

### Refinement

H atoms were positioned geometrically and refined using a riding model with C—H = 0.95–0.99 Å and with *U*_iso_(H) = 1.2 (1.5 for methyl groups) times *U*_eq_(C).

### Crystal data

**Table:**

C_16_H_18_O_3_S	*D*_x_ = 1.263 Mg m^−^^3^
*M**_r_* = 290.36	Melting point: 400 K
Orthorhombic, *P*2_1_2_1_2_1_	Mo *K*α radiation, λ = 0.71073 Å
Hall symbol: P 2ac 2ab	Cell parameters from 9922 reflections
*a* = 7.4170 (6) Å	θ = 2.5–27.6°
*b* = 8.5406 (7) Å	µ = 0.22 mm^−^^1^
*c* = 24.0980 (19) Å	*T* = 298 K
*V* = 1526.5 (2) Å^3^	Prism, colourless
*Z* = 4	0.38 × 0.37 × 0.31 mm
*F*(000) = 616	

### Data collection

**Table:**

Bruker SMART APEX CCD diffractometer	3645 independent reflections
Radiation source: fine-focus sealed tube	3380 reflections with *I* > 2σ(*I*)
graphite	*R*_int_ = 0.028
Detector resolution: 0.661 pixels mm^-1^	θ_max_ = 27.9°, θ_min_ = 1.7°
ω scans	*h* = −9→9
Absorption correction: multi-scan (*SADABS*; Sheldrick, 2000)	*k* = −11→11
*T*_min_ = 0.922, *T*_max_ = 0.942	*l* = −31→31
20418 measured reflections	

### Refinement

**Table:**

Refinement on *F*^2^	Secondary atom site location: difference Fourier map
Least-squares matrix: full	Hydrogen site location: inferred from neighbouring sites
*R*[*F*^2^ > 2σ(*F*^2^)] = 0.039	H-atom parameters not refined
*wR*(*F*^2^) = 0.099	*w* = 1/[σ^2^(*F*_o_^2^) + (0.0693*P*)^2^ + 0.0141*P*] where *P* = (*F*_o_^2^ + 2*F*_c_^2^)/3
*S* = 1.06	(Δ/σ)_max_ < 0.001
3645 reflections	Δρ_max_ = 0.25 e Å^−^^3^
185 parameters	Δρ_min_ = −0.13 e Å^−^^3^
0 restraints	Absolute structure: Flack (1983), 1534 Friedel pairs
Primary atom site location: structure-invariant direct methods	Flack parameter: 0.01 (6)

### Special details

**Table:**

Geometry. All e.s.d.'s (except the e.s.d. in the dihedral angle between two l.s. planes) are estimated using the full covariance matrix. The cell e.s.d.'s are taken into account individually in the estimation of e.s.d.'s in distances, angles and torsion angles; correlations between e.s.d.'s in cell parameters are only used when they are defined by crystal symmetry. An approximate (isotropic) treatment of cell e.s.d.'s is used for estimating e.s.d.'s involving l.s. planes.
Refinement. Refinement of *F*^2^ against ALL reflections. The weighted *R*-factor *wR* and goodness of fit *S* are based on *F*^2^, conventional *R*-factors *R* are based on *F*, with *F* set to zero for negative *F*^2^. The threshold expression of *F*^2^ > σ(*F*^2^) is used only for calculating *R*-factors(gt) *etc*. and is not relevant to the choice of reflections for refinement. *R*-factors based on *F*^2^ are statistically about twice as large as those based on *F*, and *R*- factors based on ALL data will be even larger.

### Fractional atomic coordinates and isotropic or equivalent isotropic displacement parameters (Å^2^)

**Table:**

	*x*	*y*	*z*	*U*_iso_*/*U*_eq_	
S1	0.57970 (5)	0.27901 (5)	0.149352 (15)	0.04842 (12)	
O1	0.77239 (19)	0.07949 (18)	0.33551 (5)	0.0664 (4)	
O2	0.57075 (15)	0.25318 (11)	0.26763 (4)	0.0500 (3)	
O3	0.6876 (2)	0.39213 (16)	0.11616 (5)	0.0671 (4)	
C1	0.8129 (2)	0.0711 (2)	0.28050 (7)	0.0527 (4)	
C2	0.7012 (2)	0.15921 (17)	0.24538 (6)	0.0447 (3)	
C3	0.73064 (19)	0.15714 (18)	0.18847 (6)	0.0451 (3)	
C4	0.8742 (2)	0.0726 (2)	0.16503 (7)	0.0544 (4)	
C5	0.9821 (2)	−0.0114 (2)	0.20084 (9)	0.0621 (4)	
H5	1.0779	−0.0684	0.1863	0.074*	
C6	0.9531 (2)	−0.0143 (2)	0.25777 (8)	0.0621 (4)	
H6	1.0278	−0.0736	0.2806	0.074*	
C7	0.9144 (3)	0.0710 (3)	0.10365 (8)	0.0695 (5)	
H7A	1.0391	0.0458	0.0979	0.104*	
H7B	0.8892	0.1723	0.0882	0.104*	
H7C	0.8403	−0.0062	0.0858	0.104*	
C8	0.8723 (4)	−0.0178 (4)	0.37212 (9)	0.0968 (8)	
H8A	0.8235	−0.0095	0.4089	0.145*	
H8B	0.9962	0.0149	0.3724	0.145*	
H8C	0.8649	−0.1245	0.3598	0.145*	
C9	0.4070 (3)	0.1761 (2)	0.28212 (10)	0.0709 (5)	
H9A	0.3163	0.2528	0.2903	0.106*	
H9B	0.4267	0.1114	0.3141	0.106*	
H9C	0.3679	0.1122	0.2517	0.106*	
C10	0.49336 (19)	0.13539 (18)	0.10183 (6)	0.0454 (3)	
C11	0.4650 (3)	0.1815 (2)	0.04729 (7)	0.0563 (4)	
H11	0.4975	0.2815	0.0358	0.068*	
C12	0.3884 (3)	0.0780 (2)	0.01049 (7)	0.0613 (4)	
H12	0.3698	0.1089	−0.0261	0.074*	
C13	0.3386 (2)	−0.0713 (2)	0.02686 (7)	0.0588 (4)	
C14	0.3663 (2)	−0.1131 (2)	0.08149 (8)	0.0580 (4)	
H14	0.3330	−0.2128	0.0931	0.070*	
C15	0.4417 (2)	−0.01158 (18)	0.11920 (6)	0.0511 (3)	
H15	0.4577	−0.0418	0.1560	0.061*	
C16	0.2546 (4)	−0.1816 (3)	−0.01457 (11)	0.0911 (8)	
H16A	0.3326	−0.1919	−0.0462	0.137*	
H16B	0.1401	−0.1410	−0.0263	0.137*	
H16C	0.2378	−0.2824	0.0023	0.137*	

### Atomic displacement parameters (Å^2^)

**Table:**

	*U*^11^	*U*^22^	*U*^33^	*U*^12^	*U*^13^	*U*^23^
S1	0.0537 (2)	0.04504 (19)	0.04650 (18)	0.00360 (16)	0.00099 (15)	−0.00251 (14)
O1	0.0699 (8)	0.0764 (9)	0.0528 (6)	0.0157 (7)	−0.0092 (5)	0.0025 (6)
O2	0.0525 (6)	0.0466 (6)	0.0509 (5)	0.0076 (5)	0.0038 (5)	−0.0048 (4)
O3	0.0840 (9)	0.0527 (7)	0.0645 (7)	−0.0127 (6)	0.0057 (7)	0.0006 (6)
C1	0.0492 (8)	0.0512 (9)	0.0576 (9)	−0.0011 (7)	−0.0075 (7)	−0.0026 (7)
C2	0.0422 (7)	0.0406 (7)	0.0513 (8)	−0.0022 (6)	−0.0032 (6)	−0.0052 (6)
C3	0.0405 (7)	0.0431 (7)	0.0518 (8)	−0.0004 (6)	−0.0019 (6)	−0.0059 (6)
C4	0.0432 (7)	0.0558 (9)	0.0642 (9)	−0.0004 (7)	0.0034 (6)	−0.0108 (8)
C5	0.0406 (7)	0.0647 (10)	0.0808 (12)	0.0102 (7)	0.0022 (8)	−0.0110 (9)
C6	0.0455 (9)	0.0640 (10)	0.0766 (11)	0.0082 (8)	−0.0098 (8)	−0.0002 (9)
C7	0.0604 (10)	0.0814 (13)	0.0667 (10)	0.0081 (10)	0.0142 (9)	−0.0172 (9)
C8	0.1024 (18)	0.120 (2)	0.0680 (12)	0.0358 (17)	−0.0160 (12)	0.0157 (14)
C9	0.0540 (9)	0.0672 (11)	0.0915 (14)	0.0034 (8)	0.0160 (9)	−0.0008 (10)
C10	0.0431 (7)	0.0479 (8)	0.0452 (7)	0.0019 (6)	0.0009 (6)	−0.0012 (6)
C11	0.0641 (10)	0.0556 (9)	0.0493 (8)	−0.0001 (8)	−0.0012 (7)	0.0050 (7)
C12	0.0646 (11)	0.0735 (11)	0.0457 (7)	0.0000 (9)	−0.0038 (7)	−0.0004 (8)
C13	0.0483 (8)	0.0698 (11)	0.0583 (9)	−0.0012 (8)	−0.0009 (7)	−0.0117 (8)
C14	0.0550 (9)	0.0525 (9)	0.0665 (9)	−0.0093 (7)	0.0023 (7)	0.0003 (7)
C15	0.0505 (8)	0.0536 (8)	0.0490 (7)	−0.0021 (7)	0.0000 (6)	0.0049 (6)
C16	0.0964 (17)	0.0960 (17)	0.0808 (14)	−0.0243 (15)	−0.0147 (13)	−0.0228 (13)

### Geometric parameters (Å, °)

**Table:**

S1—O3	1.4877 (13)	C8—H8A	0.9600
S1—C3	1.7959 (15)	C8—H8B	0.9600
S1—C10	1.7961 (15)	C8—H8C	0.9600
S1—O2	2.8595 (11)	C9—H9A	0.9600
O1—C1	1.361 (2)	C9—H9B	0.9600
O1—C8	1.421 (2)	C9—H9C	0.9600
O2—C2	1.3665 (18)	C10—C15	1.377 (2)
O2—C9	1.425 (2)	C10—C11	1.388 (2)
C1—C6	1.384 (2)	C11—C12	1.375 (2)
C1—C2	1.403 (2)	C11—H11	0.9300
C2—C3	1.389 (2)	C12—C13	1.385 (3)
C3—C4	1.405 (2)	C12—H12	0.9300
C4—C5	1.378 (3)	C13—C14	1.380 (3)
C4—C7	1.509 (3)	C13—C16	1.508 (3)
C5—C6	1.389 (3)	C14—C15	1.375 (2)
C5—H5	0.9300	C14—H14	0.9300
C6—H6	0.9300	C15—H15	0.9300
C7—H7A	0.9600	C16—H16A	0.9600
C7—H7B	0.9600	C16—H16B	0.9600
C7—H7C	0.9600	C16—H16C	0.9600
			
O3—S1—C3	108.86 (8)	O1—C8—H8B	109.5
O3—S1—C10	107.00 (7)	H8A—C8—H8B	109.5
C3—S1—C10	99.27 (7)	O1—C8—H8C	109.5
O3—S1—O2	126.76 (6)	H8A—C8—H8C	109.5
C3—S1—O2	56.39 (5)	H8B—C8—H8C	109.5
C10—S1—O2	125.07 (5)	O2—C9—H9A	109.5
C1—O1—C8	117.33 (16)	O2—C9—H9B	109.5
C2—O2—C9	115.36 (12)	H9A—C9—H9B	109.5
C2—O2—S1	68.76 (8)	O2—C9—H9C	109.5
C9—O2—S1	107.45 (11)	H9A—C9—H9C	109.5
O1—C1—C6	125.38 (15)	H9B—C9—H9C	109.5
O1—C1—C2	115.42 (15)	C15—C10—C11	120.28 (15)
C6—C1—C2	119.19 (16)	C15—C10—S1	121.85 (11)
O2—C2—C3	120.42 (13)	C11—C10—S1	117.64 (13)
O2—C2—C1	119.74 (14)	C12—C11—C10	119.40 (16)
C3—C2—C1	119.74 (14)	C12—C11—H11	120.3
C2—C3—C4	121.52 (14)	C10—C11—H11	120.3
C2—C3—S1	114.39 (11)	C11—C12—C13	121.22 (15)
C4—C3—S1	123.99 (12)	C11—C12—H12	119.4
C5—C4—C3	117.11 (16)	C13—C12—H12	119.4
C5—C4—C7	119.62 (16)	C14—C13—C12	118.07 (16)
C3—C4—C7	123.27 (16)	C14—C13—C16	122.10 (19)
C4—C5—C6	122.54 (15)	C12—C13—C16	119.83 (18)
C4—C5—H5	118.7	C15—C14—C13	121.86 (16)
C6—C5—H5	118.7	C15—C14—H14	119.1
C1—C6—C5	119.86 (16)	C13—C14—H14	119.1
C1—C6—H6	120.1	C14—C15—C10	119.14 (14)
C5—C6—H6	120.1	C14—C15—H15	120.4
C4—C7—H7A	109.5	C10—C15—H15	120.4
C4—C7—H7B	109.5	C13—C16—H16A	109.5
H7A—C7—H7B	109.5	C13—C16—H16B	109.5
C4—C7—H7C	109.5	H16A—C16—H16B	109.5
H7A—C7—H7C	109.5	C13—C16—H16C	109.5
H7B—C7—H7C	109.5	H16A—C16—H16C	109.5
O1—C8—H8A	109.5	H16B—C16—H16C	109.5
			
O3—S1—O2—C2	−90.50 (11)	C2—C3—C4—C5	1.6 (2)
C3—S1—O2—C2	−1.18 (9)	S1—C3—C4—C5	177.80 (13)
C10—S1—O2—C2	75.52 (10)	C2—C3—C4—C7	−178.16 (17)
O3—S1—O2—C9	158.43 (11)	S1—C3—C4—C7	−2.0 (2)
C3—S1—O2—C9	−112.24 (11)	C3—C4—C5—C6	−0.1 (3)
C10—S1—O2—C9	−35.55 (12)	C7—C4—C5—C6	179.69 (19)
C8—O1—C1—C6	−5.7 (3)	O1—C1—C6—C5	−179.05 (18)
C8—O1—C1—C2	174.92 (19)	C2—C1—C6—C5	0.3 (3)
C9—O2—C2—C3	101.36 (18)	C4—C5—C6—C1	−0.9 (3)
S1—O2—C2—C3	1.47 (11)	O3—S1—C10—C15	157.47 (13)
C9—O2—C2—C1	−82.32 (19)	C3—S1—C10—C15	44.36 (14)
S1—O2—C2—C1	177.79 (15)	O2—S1—C10—C15	−10.85 (15)
O1—C1—C2—O2	4.2 (2)	O3—S1—C10—C11	−28.07 (16)
C6—C1—C2—O2	−175.16 (14)	C3—S1—C10—C11	−141.18 (14)
O1—C1—C2—C3	−179.42 (15)	O2—S1—C10—C11	163.61 (12)
C6—C1—C2—C3	1.2 (2)	C15—C10—C11—C12	−1.4 (3)
O2—C2—C3—C4	174.15 (14)	S1—C10—C11—C12	−175.92 (14)
C1—C2—C3—C4	−2.2 (2)	C10—C11—C12—C13	0.2 (3)
O2—C2—C3—S1	−2.40 (19)	C11—C12—C13—C14	0.6 (3)
C1—C2—C3—S1	−178.71 (12)	C11—C12—C13—C16	179.9 (2)
O3—S1—C3—C2	123.34 (12)	C12—C13—C14—C15	−0.3 (3)
C10—S1—C3—C2	−125.01 (12)	C16—C13—C14—C15	−179.6 (2)
O2—S1—C3—C2	1.19 (9)	C13—C14—C15—C10	−0.9 (3)
O3—S1—C3—C4	−53.11 (15)	C11—C10—C15—C14	1.7 (2)
C10—S1—C3—C4	58.54 (14)	S1—C10—C15—C14	176.01 (13)
O2—S1—C3—C4	−175.27 (16)		

### Hydrogen-bond geometry (Å, °)

**Table:**

*D*—H···*A*	*D*—H	H···*A*	*D*···*A*	*D*—H···*A*
C8—H8B···O3^i^	0.96	2.58	3.366 (3)	139.
C14—H14···O1^ii^	0.93	2.59	3.457 (2)	155.
C15—H15···O2^ii^	0.93	2.55	3.3886 (18)	150.
C12—H12···O3^iii^	0.93	2.56	3.406 (2)	152.

Symmetry codes: (i) −*x*+2, *y*−1/2, −*z*+1/2; (ii) −*x*+1, *y*−1/2, −*z*+1/2; (iii) *x*−1/2, −*y*+1/2, −*z*.
